# ﻿Slugs and semi-slugs of the superfamily Helicarionoidea (Gastropoda, Stylommatophora) collected in North Vietnam during the 2023 Bulgarian Zoological Expedition, with emphasis on the genus *Ostracolethe*

**DOI:** 10.3897/zookeys.1249.155684

**Published:** 2025-08-25

**Authors:** Ivailo Dedov, Quang Manh Vu, Tuan Trieu Anh

**Affiliations:** 1 Institute of Biodiversity and Ecosystem Research, Bulgarian Academy of Sciences, 2 Gagarin Street, 1113 Sofia, Bulgaria Institute of Biodiversity and Ecosystem Research, Bulgarian Academy of Sciences Sofia Bulgaria; 2 Hoa Binh University (HBU), Nam Tu Liem, Hanoi, Vietnam Hoa Binh University (HBU) Hanoi Vietnam; 3 Hung Vuong University, Nong Trang ward, Viet Tri City, Phu tho, Vietnam Hung Vuong University Viet Tri City Vietnam

**Keywords:** Biodiversity, determination key, land gastropods, new species, Southeast Asia, taxonomy, unusual anatomical structures

## Abstract

A list of species of slugs and semi-slugs of the superfamily Helicarionoidea collected in North Vietnam in 2023 is presented. A new species of the genus *Ostracolethe* Simroth, 1901, *Ostracolethe penevi* Dedov & Vu, **sp. nov.**, is described, which was collected from a single locality in North Vietnam. The validity of *Ostracolethe fruhstorfferi* Simroth, 1901 is confirmed, and the unusual torus-toroid structure is discussed. A key for the determination of the helicarionoid genera of slugs and semi-slugs from North Vietnam is also provided.

## ﻿Introduction

There are two major challenges when working with tropical slugs and especially semi-slugs. First, numerous species in older works are described only by the shell, with little or no information about the anatomy, body pattern, coloration, and size (Páll-Gergely 2019). This is a big challenge when working with especially the genus *Megaustenia* (Haines 1858; Gould 1859; Morelet 1865, 1866; Fischer 1898; Collinge 1901; Bavay and Dautzenberg 1909). The second problem is that in older publications the names of the localities (e.g. “Cochinchina”, “Annam”, and “Tonkin”) indicate very large areas, oftentimes falling within the borders of two or more countries today, which in practice means that the type locality of the species cannot be localized (Schileyko 2011). In the most recent compilation on Vietnamese pulmonate gastropods (Schileyko 2011), 477 species and subspecies from 20 families are given, of which only 20 species (of four families: Ariophantidae, Helicarionidae, Ostracolethidae, Philomycidae) belong to the slugs or semi-slugs. Compared to the extremely rich tropical fauna, the 20 species mentioned illustrate well the underestimation of slugs and semi-slugs. One of the reasons for the small number of slug and semi-slug species in Vietnam is their biology. Most often, specimens from this group are found high on the branches and leaves in the tree crowns and are difficult to reach (personal observation).

Following the summarizing work of Schileyko (2011), a couple of new species from the superfamily Helicarionoidea have been reported for the Vietnamese fauna. Schileyko (2016) published on two species collected in Central Vietnam: *Parmarion martensi* Simroth, 1894 and *Parmarion pupillaris* Humbert, 1864. Dedov et al. (2019) published on a new species from the region of Fansipan peak: *Laocaia simovi* Dedov & Schneppat, 2019, and Dedov (2024) discovered an additional new species from Tam Dao National Park: *Muangnua vumanhi* Dedov, 2024.

The aims of this article are to present the species of the superfamily Helicarionoidea collected by the Bulgarian expedition in Vietnam in 2023, to provide new information about the genus *Ostracolethe* Simroth, 1901, to describe a species new to science, and to draw the attention of the scientific community to unusual structures in tropical gastropods in the hope of provoking new research into these phenomena.

## ﻿Materials and methods

During our survey in North Vietnam in 2023, samples were hand collected in undisturbed or slightly disturbed submontane tropical forests at the beginning of the dry season (October 16–23). All specimens were found on the leaves in the bushes, the crowns of the trees, under or on tree bark, or inside moist, decaying tree trunks. Animals were photographed in situ using an Olympus Tough TG-5 digital camera. After photographic documentation in situ, specimens were processed following the methods by Nitz et al. (2009). Specimens were relaxed in a jar with water and some drops of the surfactant SUPRALAN-UF until fully extended and dead. The dead semi-slug was cleaned of mucus in a sieve under cold, running water. For preservation, 96% ethanol was injected with a syringe into the body cavity through the terminal tip of the sole. The specimen was then placed in ethanol (96%) and left for some hours. It was stored in 70% ethanol. The anatomical and morphological examinations, as well as photographs of internal body structures, were carried out with a Zeiss Discovery V12 stereomicroscope and Jenoptik Gryphax camera. The torus structures were additionally studied under a Zeiss Axio Imager M2 compound microscope and photographed with a Jenoptik Gryphax camera. The samples have been deposited in the invertebrate collection of the Institute of Biodiversity and Ecosystem Research, Bulgarian Academy of Sciences (IBER-BAS).

The torus-toroid structure of *O. fruhstorfferi* was colored following the in toto staining method of Georgiev et al. (1986).

## ﻿Results

**Phylum Mollusca**

**Class Gastropoda**

**Subclass Heterobranchia**

**Infraclass Euthyneura**

**Subterclass Tectipleura**

**Superorder Eupulmonata**

**Order Stylommatophora**

**Suborder Helicina**

**Infraorder Limacoidei**

**Superfamily Helicarionoidea**

**Family Ariophantidae**

**Subfamily Ariophantinae**

### ﻿Genus Megaustenia

Taxon classificationAnimaliaStylommatophoraAriophantidae

T.D.A. Cockerell, 1913

9178EF8E-E27E-51DD-8E9F-18034EC309A2

#### Type species.

*Vitrina praestans* A. Gould, 1843: 140; by typification of replaced name. Nom. nov. pro *Cryptosoma* Theobald, 1857, non Milne-Edwards, 1837 (Crustacea).

### ﻿ Megaustenia cf. messageri

Taxon classificationAnimaliaStylommatophoraAriophantidae

(Bavay & Dautzenberg, 1909)

F3BABBF3-0D26-58AF-BCB0-46F1349C51FD

Fig. 1

Helicarion messageri : Bavay and Dautzenberg 1909, p. 231.
Megaustenia messageri : Schileyko 2011, p. 32.
Helicarion messageri : Hoang Ngoc Khac et al. 2012, p. 319.

#### Material examined.

Vietnam • 3 adults; Vinh Phuc Province, Tam Dao town district, tropical forest up to the town, near Buddhist temple; 21.4601°N, 105.6483°E; 1045 m alt.; 16 Oct.2023; I. Dedov, N. Simov, R. Bekchiev, M. Langurov leg.; on artificial stone wall; IBER-BAS 40602.

#### Remarks.

**Figure 1.**
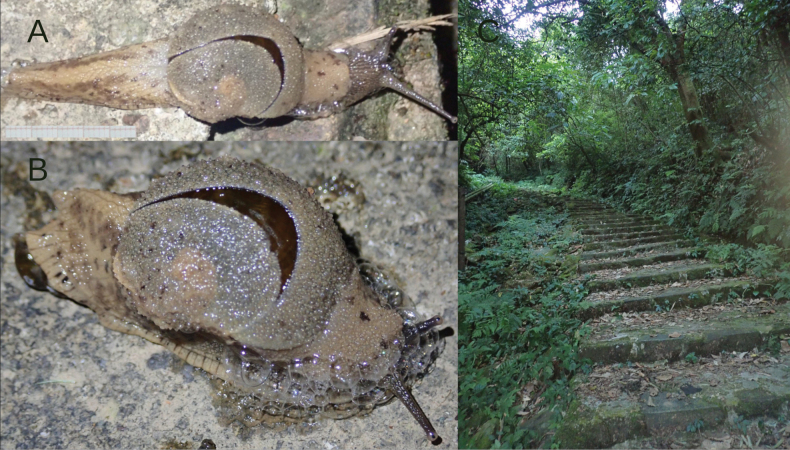
Megaustenia cf. messageri, Tam Dao National Park, IBER-BAS 40602. A. Actively crawling; B. Protective reaction to touch; C. Habitat of the species.

*Megaustenia messageri* was reported from Tam Dao National Park by Hoang Ngoc Khac et al. (2012). We found three active specimens on the soil surface (Fig. 1A, B) during the night collection in the tropical forest not far from Tam Dao town (Fig. 1C).

#### Habitat.

Slightly disturbed submontane tropical forests

#### Distribution.

North Vietnam (Schileyko 2011).

##### ﻿Subfamily Ostracolethinae

### ﻿Genus Ostracolethe

Taxon classificationAnimaliaStylommatophoraAriophantidae

Simroth, 1901

13FC345F-503A-55DF-843D-E2F3013B1A29

#### Type species.

*Ostracolethe fruhstorfferi* Simroth, 1901: 64.

#### Remarks.

*Ostracolethe* is a subjective synonym of *Myotesta* Collinge, 1901, which was published on the same day. Hoffmann (1924: 378) acted as first reviser and determined the precedence of *Ostracolethe* over *Myotesta*.

### ﻿ Ostracolethe fruhstorfferi

Taxon classificationAnimaliaStylommatophoraAriophantidae

Simroth, 1901

65B1E911-1218-5D27-A3E5-ECFFE074B08D

**Figure 2.**
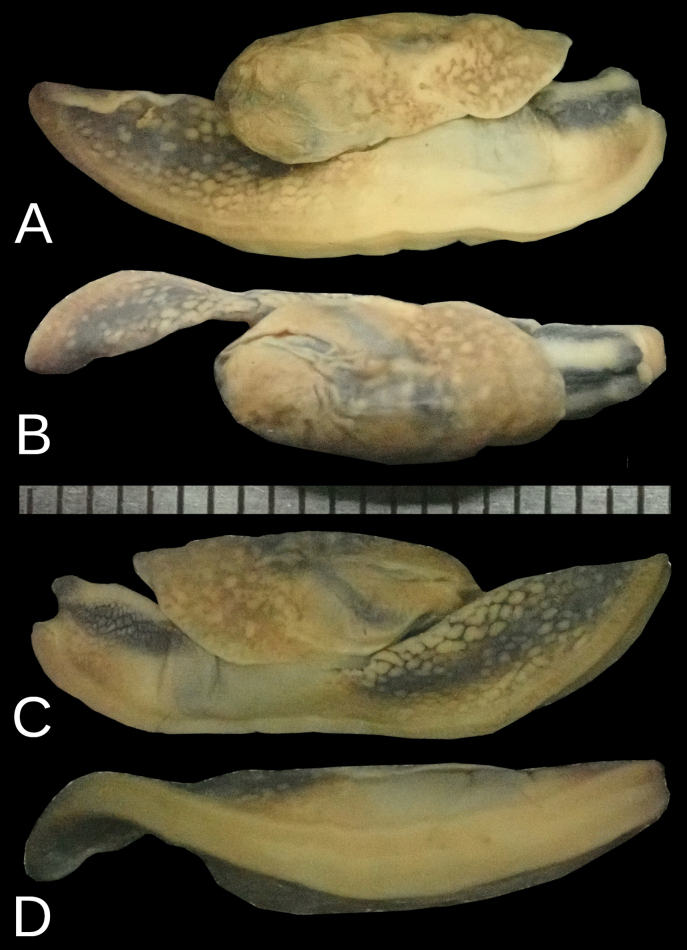
*Ostracolethe fruhstorfferi*, fixed animal, IBER-BAS 40616. A. Right side; B. Top; C. Left side; D. Sole.

**Figure 3.**
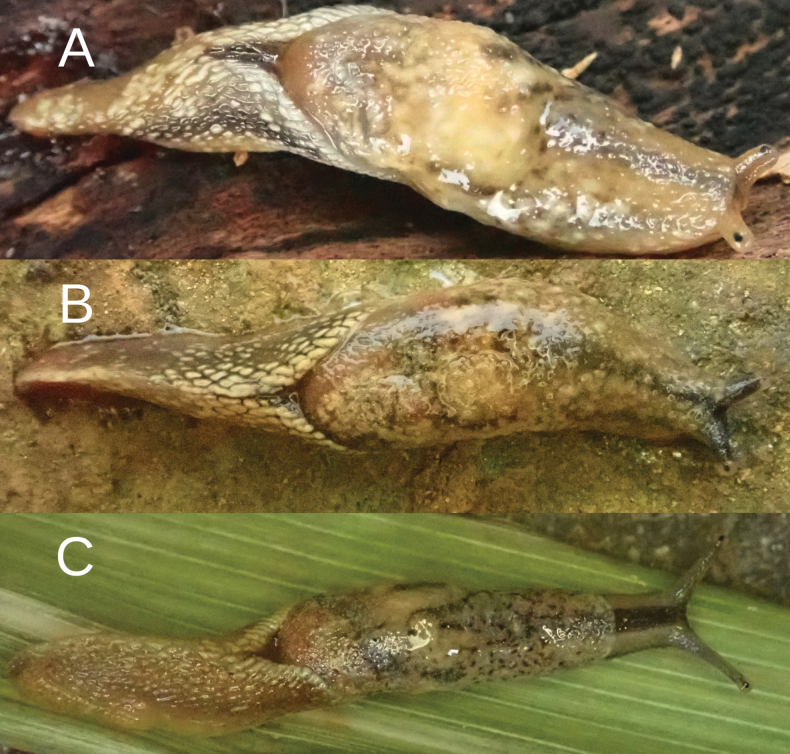
*Ostracolethe fruhstorfferi*, live animals. A. Vinh Phuc Province, Tam Dao National Park, IBER-BAS 40604; B. Lào Cai Province, Ngu Chi Son village, IBER-BAS 40616; C. *O.* (?) *penevi* Dedov & Vu, sp. nov., Lào Cai Province, Thac Bac, IBER-BAS 40618.

**Figure 4.**
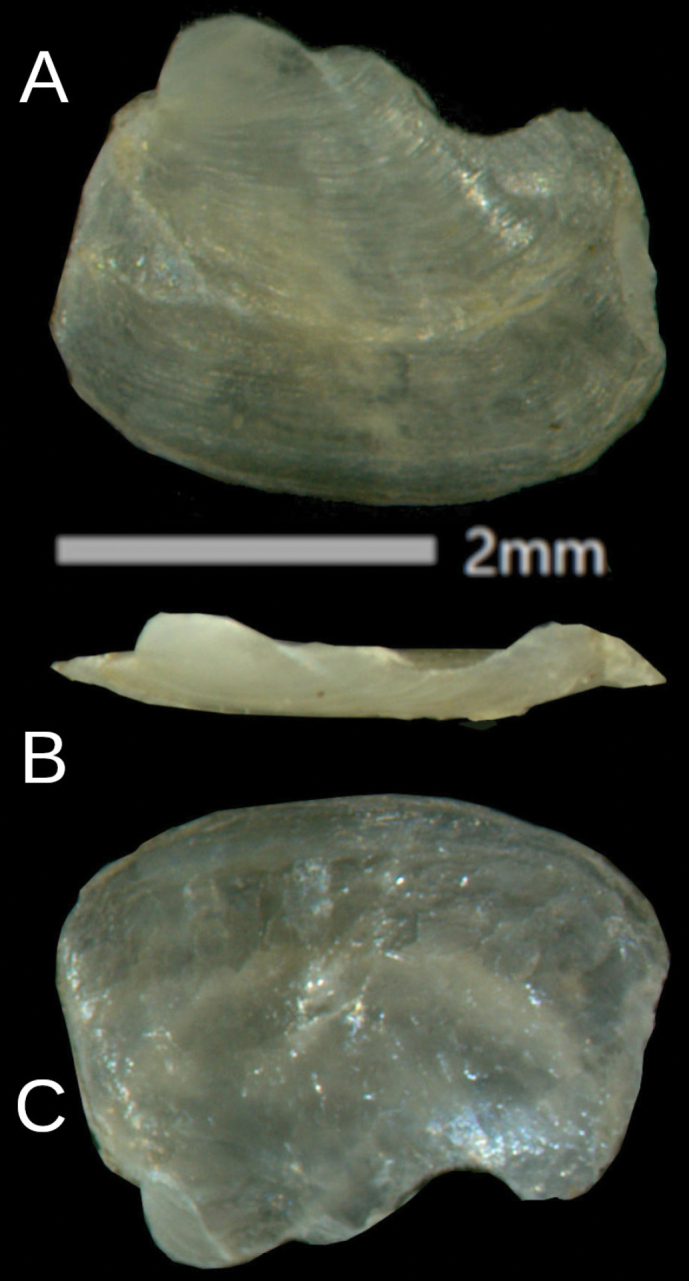
*Ostracolethe fruhstorfferi*, shell, IBER-BAS 40616. A. Top side; B. Lateral view; C. Underside side.

**Figure 5.**
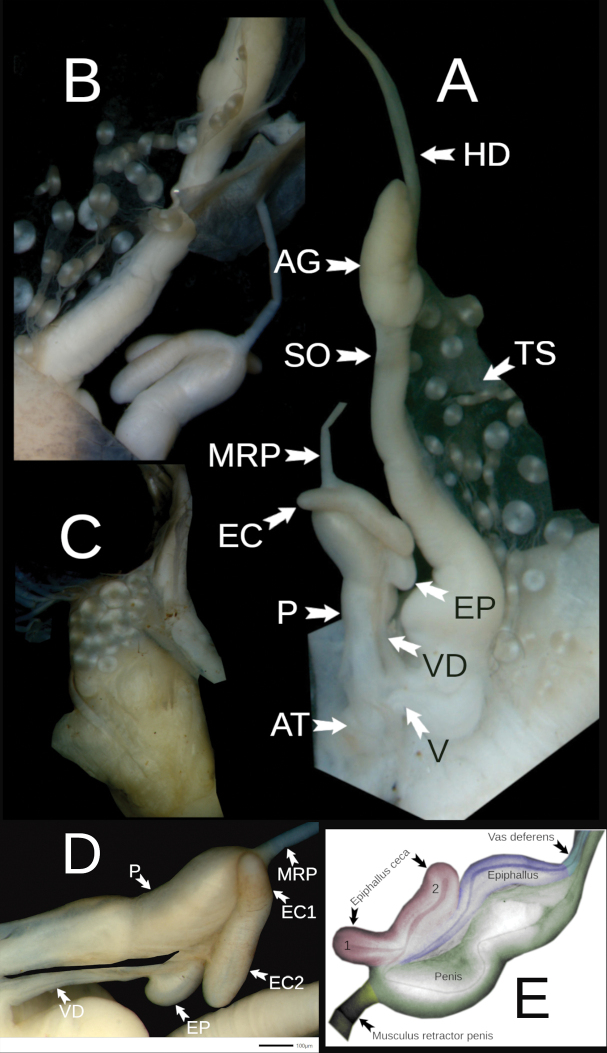
*Ostracolethe fruhstorfferi*, anatomy. A. General view of the reproductive system, IBER-BAS 40616; B. Penis, view from the side of body integuments, IBER-BAS 40616; C. In situ position of the tori bunch, IBER-BAS 40604; D. Penis, view from the side of viscera, IBER-BAS 40616; E. Diagram of the structure of the male part of reproductive system, base on IBER-BAS 40604, greenish–penis, gray-yellowish–musculus retractor penis, violet–epiphallus, pinkish–epiphallus ceca (1 and 2), bluish–vas deferens. Abbreviations: AT–atrium, P–penis, MRP–musculus retractor penis, EP–epiphallus, EC–epiphallus ceca, VD–vas deferens, V–vagina, SOD–spermoviduct, AG–albumen gland, HD–hermaphrodite duct, TS–torus-toroid structures.

**Figure 6.**
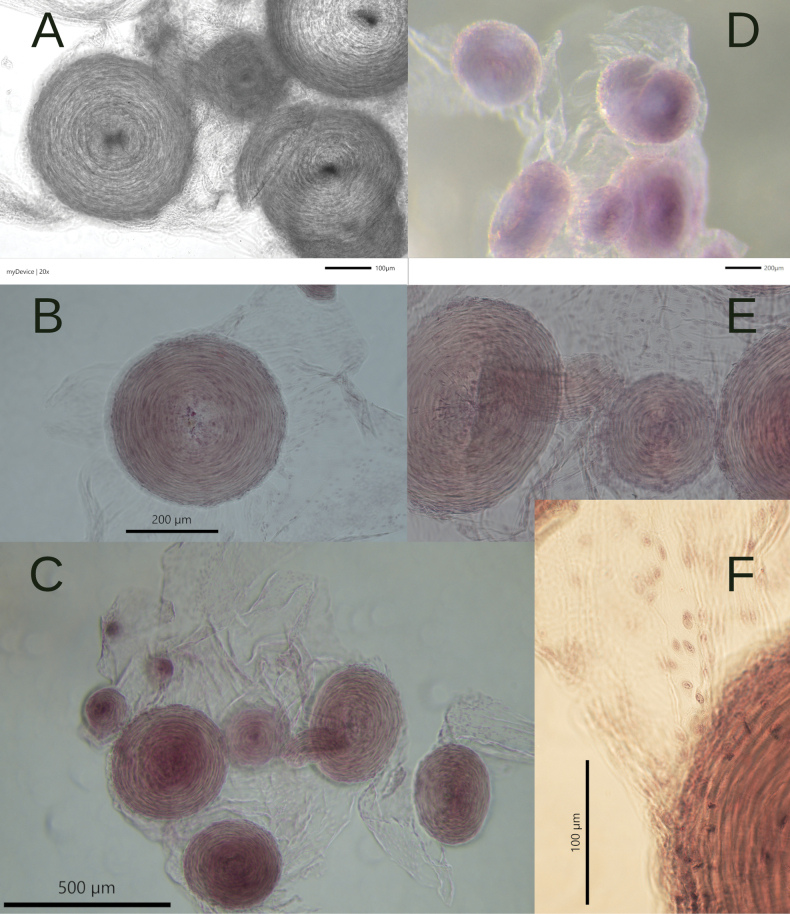
*Ostracolethe fruhstorfferi*, torus-toroid structure. A. Natural in ethanol, under stereomicroscope B, C, E, F. Colored, under microscope in different magnification; D. Colored, in ethanol, under stereomicroscope.

**Figure 7.**
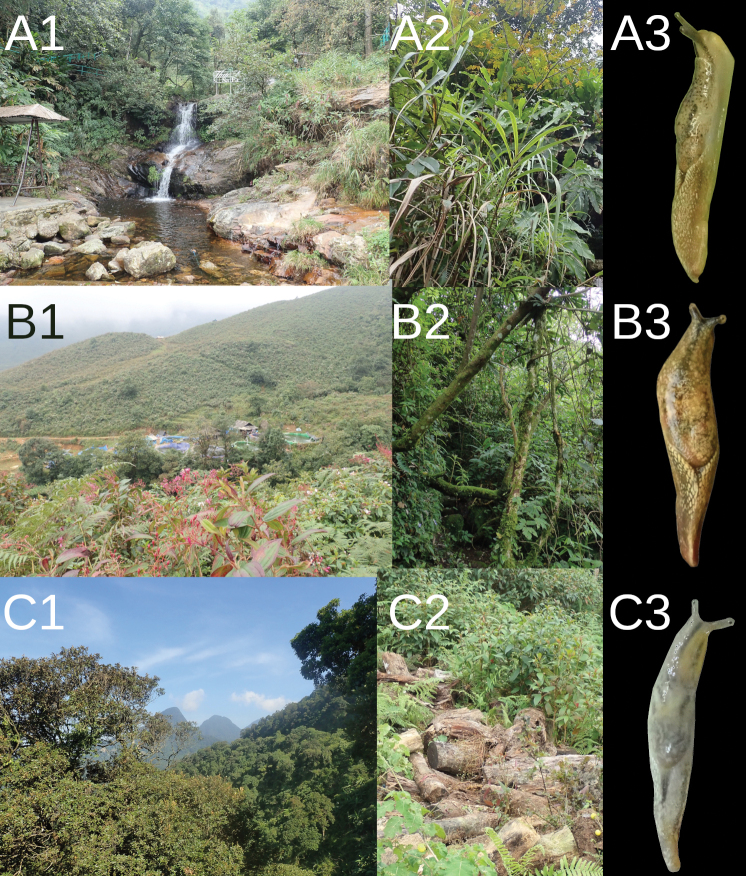
Localities of genus *Ostracolethe*. A. *O.* (?) *penevi* Dedov & Vu, sp. nov., Lào Cai Province, Thac Bac, IBER-BAS 40618, A1–landscape view, A2–habitat, A3–specimens; B. *O. fruhstorfferi*, Lào Cai Province, Ngu Chi Son village, IBER-BAS 40616, B1–landscape view, B2–habitat, B3–specimens; C. *O. fruhstorfferi*, Vinh Phuc Province, Tam Dao National Park, IBER-BAS 40604, C1–landscape view, C2–habitat, C3–specimens. The photos are arranged from bottom to top according to altitude at each locality.

**Figure 8.**
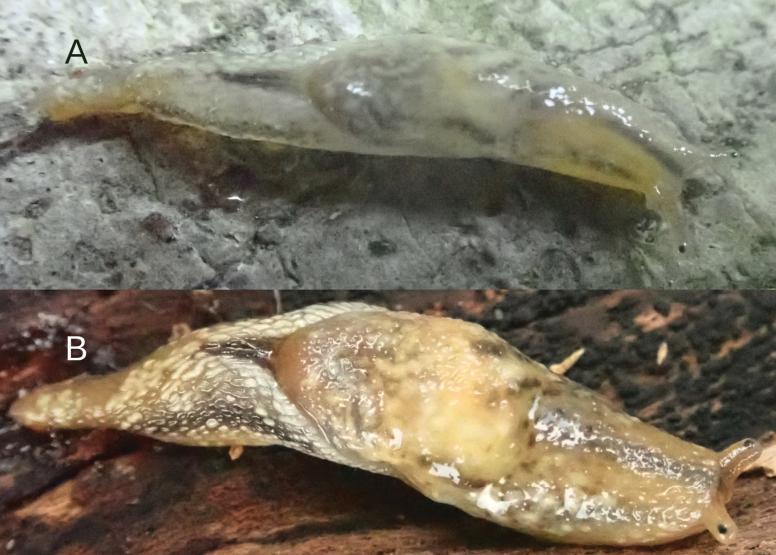
*Ostracolethe fruhstorfferi*, very same specimen, IBER-BAS 40604. A. On rock surface; B. On tree bark.

Figs 2 , 3A, B , 4 , 5 , 6 , 7 , 8

Ostracolethe fruhstorfferi : Simroth 1901, p. 64.
Myotesta fruhstorferi : Collinge 1901, p. 118.
Myotesta fruhstorferi : Fischer 1904, Dautzenberg, p. 8.
Ostracolethe fruhstorfferi : Fischer 1904, Dautzenberg, p. 9.
Ostracolethe fruhstorfferi : Schileyko 2003, p. 1340.
Ostracolethe fruhstorfferi : Schileyko 2011, p. 37.

#### Material examined.

Vietnam • 1 adult; Lào Cai Province, Sa Pa district, Ngu Chi Son village, tropical forest near village; 22.4283°N, 103.7495°E; 1505 m alt.; 22 Oct. 2023; Chi Toan Le leg.; on leaves of a tree; IBER-BAS 40616; • 1 subadult; Vinh Phuc Province, Tam Dao town, Tam Dao National Park, tropical forest near the town; 21.4672°N, 105.6440°E; 1005 m alt.; 17 Oct. 2023; I. Dedov, N. Simov, R. Bekchiev, M. Langurov leg.; under tree bark of decaying trunks; IBER-BAS 40604.

#### Description.

The body is relatively wider and shorter than *Ostracolethe penevi* Dedov & Vu, sp. nov. The visceral hump is large and elevated, but this elevation is better visible in fixed animals (Fig. 2A, C). The preserved body dimensions (*N* = 1, adult specimen) are: body length 24 mm, body width 5.2 mm, visceral hump length 11.9 mm, visceral hump width 5.2 mm, length of free mantle flap 5.2 mm, sole length 21.2 mm, sole width 2.6 mm, width of lateral zones of sole 0.75–0.85 mm, central zone of sole 0.85–0.95 mm, length of the genital pore 0.5 mm, width of the genital pore 0.6 mm (Fig. 2A–D).

The primary color of the body is pinkish-ocher-brownish (Fig. 3A, B), although variable (see Discussion). On the neck, three well-defined, blackish-gray- stripes are visible (one on the top and two laterally, best visible in fixed specimens). The tentacles are pale grayish, which are more intensely colored near the ommatophores and visible in active live animals. On the pinkish-ocher-brownish visceral hump, numerous yellowish spots are visible and grouped in larger stains and zones of irregular shapes. The posterior of the body has similar colors; yellowish pigment is grouped on the edge of the Y-shaped dorsal groove behind the visceral hump. The sole of living specimens is generally pale, but with lateral zones more pinkish in color, while the central zone is milky whitish. The differences in coloration between central and lateral zones is still visible in preserved specimens. The body cavity is not enlarged in the posterior part of the foot, but this part is not as muscular as in *Ostracolethe* sp. nov. and *Laocaia simovi* (Dedov et al. 2019). The caudal horn is not very prominent.

Shell is fingernail-like (3.2 × 2.3 mm), and sparse growth lines are visible on the upper surface. The rudimentary shell is fully covered by the mantle (Fig. 4A–C).

Reproductive system (Fig. 5A–E). The penis is relatively short and thinner in the area towards the atrium. The penis retractor muscle is apically attached to the bulbiform posterior end of the penis. The epiphallus is relatively short, with two caeca, one shorter and another longer, located opposite each other (Fig. 5D, E). The vas deferens is short and entering epiphallus laterally. The vagina is thick and short. The bursa copulatrix is not well visible and is not visible in the drawing by Schileyko (2003: fig. 1752); it is most probably closely adjacent to the spermoviduct duct, as in *O. penevi* sp. nov. The albumen is gland small.

#### Remarks.

According to Simroth (1901) (in Collinge 1902, p. 14) “the vas deferens has, before it passes into the penis, 3 short, thick flagella”. In fact, the “three tubercles” visible on the side of the body integuments (Fig. 5 B) represent the structure described above, which is better visible on the side of the viscera (Fig. 5 D, E).

The “disc” structure reported by Collinge (1902) is present (Fig. 6A–F). The “discs” are not flat but rather have a torus-toroid shape (doughnut-like) readily visible in Figure 6D. The tori are attached to a veil of the base of connective tissue at one of its flat parts. The structure consists of 35–40 tori of varying sizes (diameter 77.88–379.26 µm) (Fig. 6A–F). When viewed at high magnification under a stereomicroscope, the “torus” appears to be formed by a network of symmetrically interwoven fibers (Fig. 6A−C, E, F).

#### Habitat.

*Ostracolethe fruhstorfferi* inhabits tropical and degraded tropical forests, where it lives on leaves or branches in the crowns of trees (Fig. 7B, C).

#### Biology.

Color change to blend in with the environment, which is a new phenomenon of metachrosis among terrestrial gastropods, was observed in specimens from Tam Dao National Reserve (IBER-BAS 40604). When a specimen was photographed on a stone surface it looked whitish transparent, and only melanin kept the pattern of coloration (Fig. 8A). The very same specimen photographed on tree bark became more colorful, and pinkish, ocher, brownish, and yellowish colors appeared (Fig. 8B).

#### Distribution.

North Vietnam (Schileyko 2003).

### ﻿ Ostracolethe (?) penevi

Taxon classificationAnimaliaStylommatophoraAriophantidae

 Dedov & Vu sp. nov.

1869456B-986E-5CE2-88BF-5D1E928C1E53

https://zoobank.org/8701ABF3-E296-4C3A-809B-55EB3A3D3EAE

**Figure 9.**
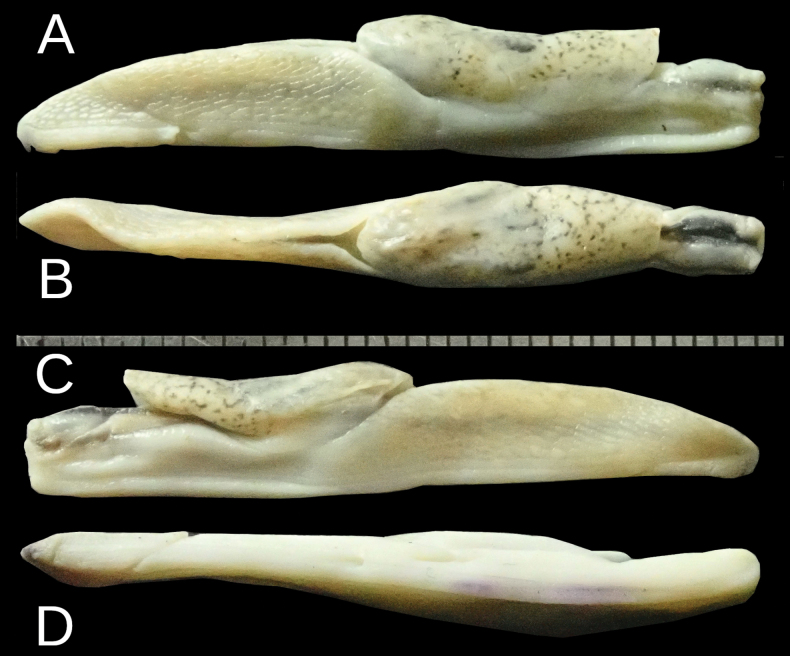
*Ostracolethe* (?) *penevi* Dedov & Vu, sp. nov., fixed animal, IBER-BAS 40618. A. Right side; B. Top; C. Left side; D. Sole.

**Figure 10.**
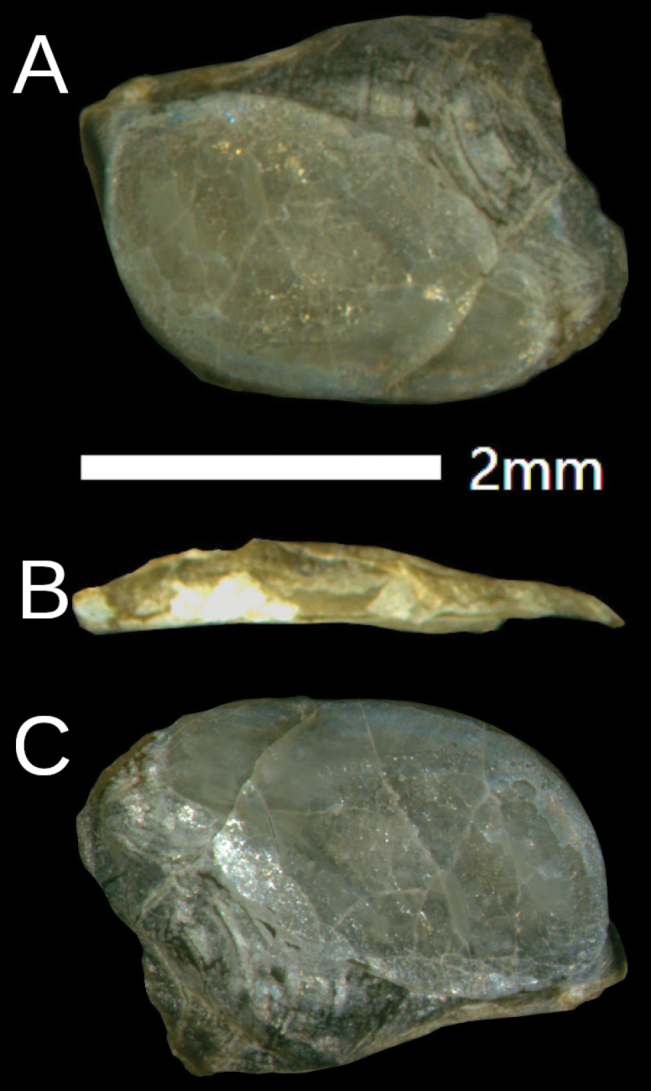
*Ostracolethe* (?) *penevi* Dedov & Vu, sp. nov., shell, IBER-BAS 40618. A. Top side; B. Lateral view; C. Underside.

**Figure 11.**
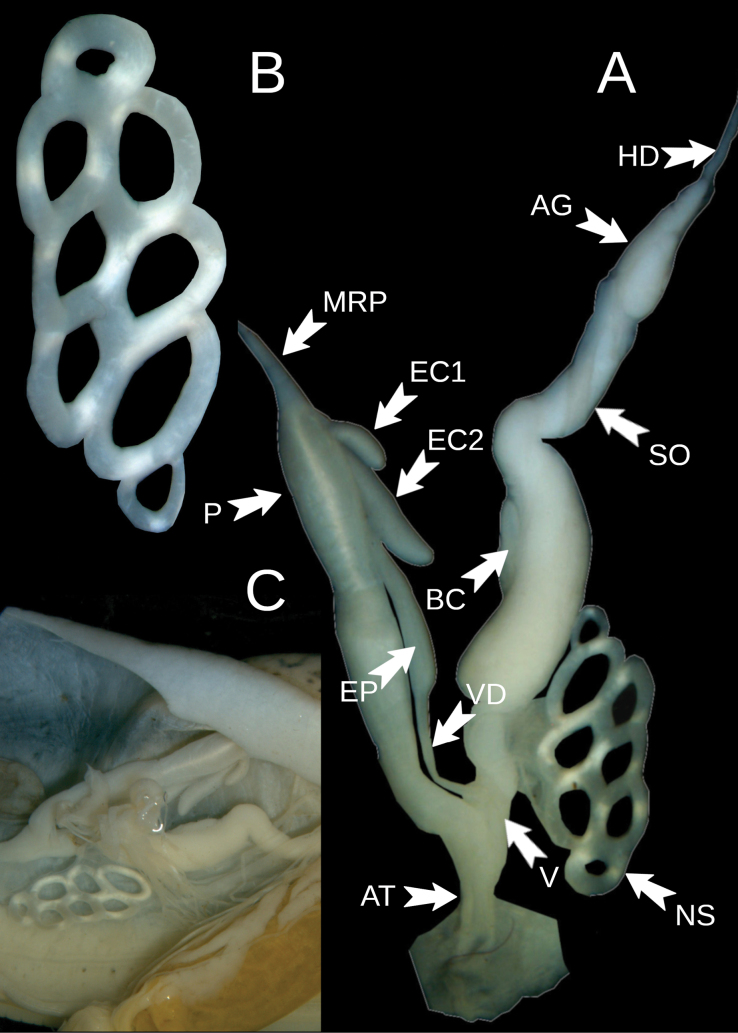
*Ostracolethe* (?) *penevi* Dedov & Vu, sp. nov., anatomy, IBER-BAS 40616. A. General view of the reproductive system: AT–atrium, P–penis, MRP–musculus retractor penis, EP–epiphallus, EC1–epiphallus ceca short, EC2–epiphallus ceca long, VD–vas deferens, BC–bursa copulatrix, V–vagina, SO–spermoviduct, AG–albumen gland, HD–hermaphrodite duct, NS–net structure; B. Net structure itself; C. In situ position of the net structure.

Figs 3C , 9 , 10 , 11

#### Material examined.

Vietnam • 1 adult; Lào Cai Province, Sa Pa district, Thac Bac (= Silver waterfall); 22.3627°N, 103.7773°E; 1677 m alt.; 23 Oct. 2023; I. Dedov, N. Simov, R. Bekchiev, M. Langurov leg.; forest around touristic area; IBER-BAS 40618.

#### Differential diagnosis.

*Ostracolethe* (?) *penevi* Dedov & Vu, sp. nov. differs from *O. fruhstorfferi* by its lighter coloration and different color pattern; longer and more slender body; slightly raised long visceral hump shifted towards the head (not central on the body); longer epiphallus; the positions of the epiphallus caeca, which are successively, one after the other (from the epiphallus to the penis, long and short), not opposite to each other as in *O. fruhstorfferi*, the vas deferens (vd) entering epiphallus slightly laterally (not clearly lateral as in *O. fruhstorfferi*), and presence of net-like structure with eight circles near the female part of reproductive system.

#### Description.

The body is gracile (Fig. 3C). The visceral hump is relatively long, slightly raised, and is displaced towards the front of the body. The preserved body dimensions (*N* = 1) are: body length 38.3 mm, body width 5.7 mm, visceral hump length 15.5 mm, visceral hump width 5.7 mm, length of free mantle flap 5.4 mm, sole length 36.3 mm, sole width 3.0 mm, width of lateral zones of sole 0.85–1.15 mm, width of central zone of sole 0.85–0.9 mm, length of the genital pore (not well visible) 0.8 mm, width of the genital pore 0.55 mm (not well visible) (Fig. 9A–D).

Body primary color is light-yellowish-ocher. On the neck, three well-defined gray-blackish stripes are visible (one on the top and two laterally). The tentacles are yellowish. On light-yellowish-ocher (slightly ocher-reddish in its posterior part) visceral hump numerous gray-blackish dots are visible, sometimes grouped in short stripes or spots with irregular shape. Posterior body almost uniformly light-yellowish-ocher with very small dots, well visible under magnification. The dorsal edge of the Y-shaped dorsal groove behind the visceral hump is yellowish. The sole in living specimens is pale-yellowish, more intense yellowish in its lateral margins. In preserved animals, the complete sole loses its pigmentation, which is also the case for all other body parts of the specimens (Fig. 3C). All colorful pigments (besides melanin) are dissolved in ethanol during the preservation. The body cavity is not enlarged into the posterior part of the foot, which is fully muscular. The caudal horn protrudes slightly beyond the contour of the body and is similarly colored.

The shell is plate-like, oval-rhomboid (3 × 2 mm), at one end with visible, and with sparse growth lines (Fig. 10A–C). The rudimentary shell is fully covered by the mantle.

Reproductive system (Fig. 11 A). The penis is long, thick-walled, and spindle-shaped but slightly pinched around the middle. The penis retractor muscle is attached apically to the posterior end of the penis. The epiphallus is long, about 2/3 of the penis length, with 2 caeca: a longer and smaller (1/3 of the longer caeca). The vas deferens is comparatively short and entering epiphallus slightly laterally. The free oviduct is relatively short. The vagina is thick and short. The bursa copulatrix is relatively long, finger-like (not divided into a bursa copulatrix and bursa copulatrix duct), closely adjacent to the spermoviduct duct, and reaching about halfway to the albumen gland. The albumen gland is small and pearly-yellowish.

An unusual net-like structure was found close to the female part of the reproductive system (Fig. 11B, C). The structure is relatively large and resembles a symmetric net-like formation with eight holes and has dimensions of 3.7 × 1.7 mm (compared to the 14.5 mm length of the reproductive system). Its function remains a mystery, but given its position, it could be homologous to the mucus glands in Helicidae. However, there are no clearly visible channel(s) between this structure and the rest of the reproductive system, even under high magnification. The structure appears to be attached to the female reproductive system by a tangle of connective tissue. The atrium is tubular.

#### Distribution.

North Vietnam, type locality.

#### Habitat.

*Ostracolethe* (?) *penevi* Dedov & Vu, sp. nov. inhabits the leaves of high herbaceous and shrubby moisture-loving vegetation (Fig. 7A).

#### Derivatio nominis.

The new species is named after Prof. Dr Lyubomir Penev for his incredible contribution to the reputation of Bulgarian science and his tireless, friendly support over the years.

##### ﻿Family Helicarionidae

**Subfamily Durgellinae**

### ﻿Genus Muangnua

Taxon classificationAnimaliaStylommatophoraHelicarionidae

Solem, 1966

35EBE80C-C64B-594E-AD85-58F7C2FA287D

#### Type species.

*Muangnua limax* Solem, 1966: 65, by original designation.

### ﻿ Muangnua vumanhi

Taxon classificationAnimaliaStylommatophoraHelicarionidae

Dedov, 2024

B843AD37-5A7D-5B00-B871-5E07408D55AC

Fig. 12

Muangnua vumanhi : Dedov 2024, p. 319–323.

#### Material examined.

Vietnam • 1 adult; Vinh Phuc Province, Tam Dao town, tropical forest up to the town; 21.4606°N, 105.6487°E; 1055 m alt.; 16 Oct. 2023; I. Dedov, N. Simov, R. Bekchiev, M. Langurov leg.; on and under tree bark with many tree-welling mushrooms, and in decaying logs; IBER-BAS 40601.

#### Remarks.

**Figure 12.**
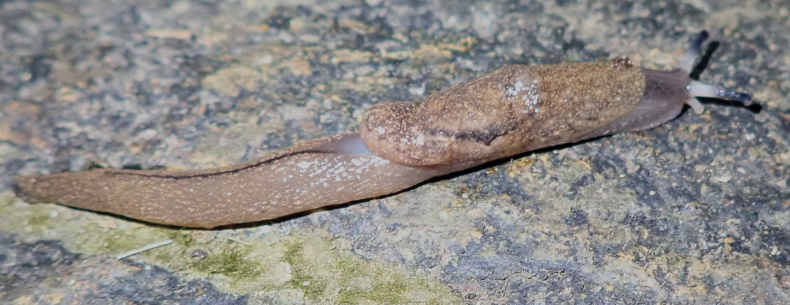
*Muangnua vumanhi*, Vinh Phuc Province, Tam Dao National Park, IBER-BAS 40601.

This species (Fig. 12) was recently reported from Tam Dao National Park by Dedov (2024).

##### ﻿Subfamily Helicarioninae

### ﻿Genus Laocaia

Taxon classificationAnimaliaStylommatophoraHelicarionidae

Kuzminykh, 1999

CB410589-CD34-5F61-AD44-FA2B1DF63394

#### Type species.

*Laocaia attenuata* Kuzminykh, 1999: 47–48, by original designation.

##### ﻿*Laocaia* (?) sp. nov.

**Figure 13.**
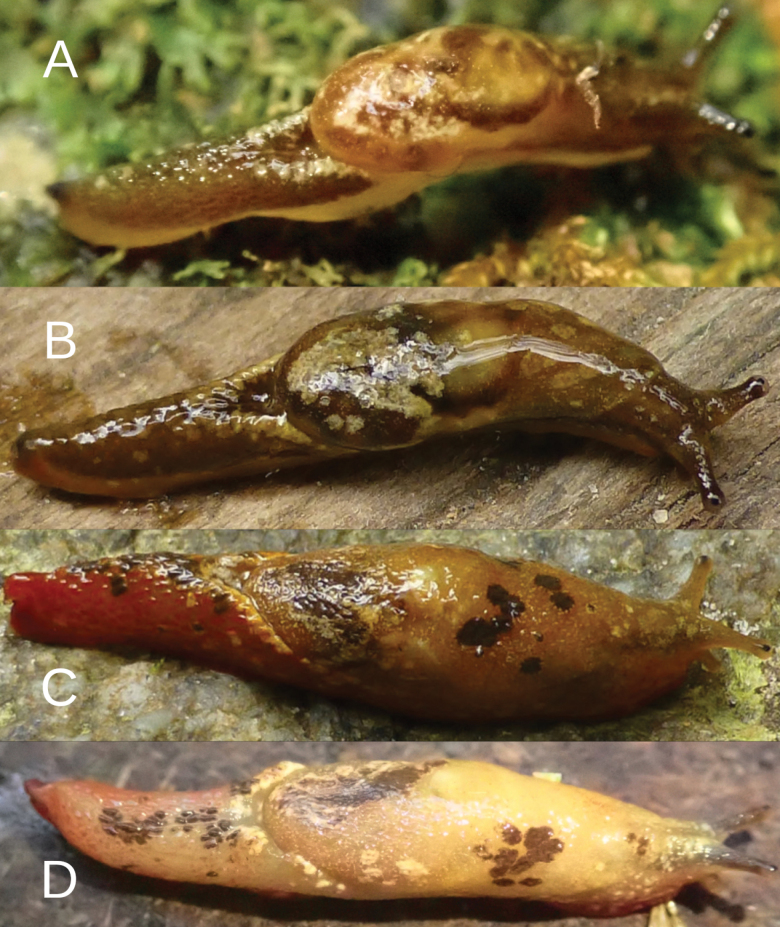
A, B. *Laocaia* (?) sp. nov., live animals, Lào Cai Province, Fansipan peak. A. 3085 m alt.; B. 3040 m alt.; C, D. *L. simovi* Dedov & Schneppat, 2019., Lào Cai Province, Fansipan peak; C. 2990 m alt.; D. 3040 m alt.

**Figure 14.**
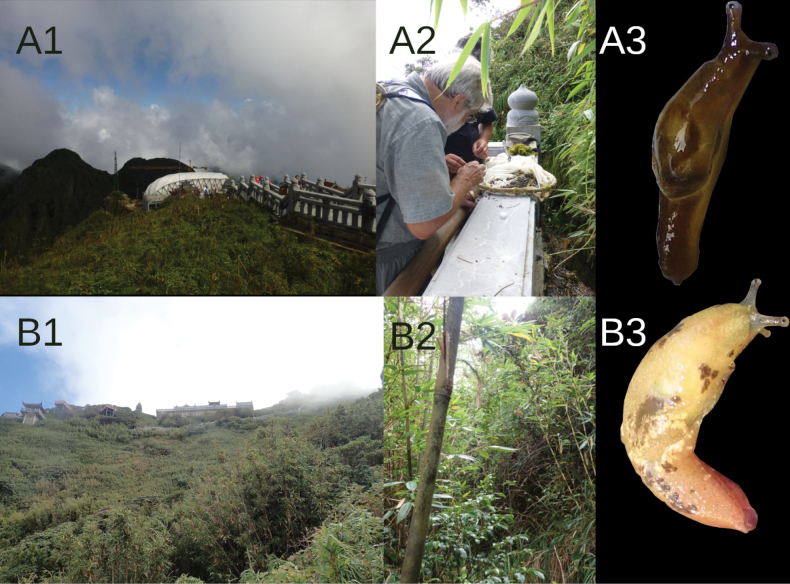
Localities of genus *Laocaia*. A. *Laocaia* sp. nov., Lào Cai Province, Fansipan peak, 3085 alt., IBER-BAS 40338, A1–landscape view, A2–habitat, A3–specimens; B. *L. simovi*, Lào Cai Province, Fansipan peak, 2990–3040 m alt., IBER-BAS 40604, B1–landscape view, B2–habitat, B3–specimens. For bottom to top, the pictures are arranged according to the altitude of the locality.

Figs 13A, B, 14A1–A3

**Material examined.** Vietnam • 1 juvenile; Lào Cai Province, Fansipan peak, nearby the peak; 22.3029°N, 103.7753°E; 3085 m alt.; 21 Oct. 2018; leg. I. Dedov, N. Simov, R. Bekchiev, P. Beron; rocks; IBER-BAS 40338 • 2 juveniles; Sa Pa district, Fansipan peak, bamboo forest below the peak; 22.3046°N, 103.7759°E; 3040 m alt.; 24 Oct. 2023; I. Dedov, N. Simov, R. Bekchiev, M. Langurov leg.; IBER-BAS 40619-2.

**Remarks.** Almost at the top of Mount Fansipan (3040 m a.s.l.) (Fig. 14A) in 2018 we found a juvenile specimen of an unknown slug, possibly belonging to the genus *Laocaia* (Fig. 13A). We searched the area of the peak again in 2023 and found two more juvenile specimens (Fig. 13B) at low altitude in the very same spot as the yellowish morph of *L. simovi* (Fig. 14B). The specimens have a typical pattern different from all other *Laocaia* species, as well as from the slugs known from the province Lào Cai (Kuzminykh 1999; Dedov et al. 2019; Đỗ Đức Sáng et al. 2022; Dedov 2024).

The anterior part of the body is primarily dark-ocher-brownish with horizontal yellowish belt-zone on the underside. On the anterior part of the body, there are also additional whitish, irregular spots and markings. The head and tentacles are dark-brownish, with numerous small white markings on the neck. The visceral hump is dark-ocher-brownish, with irregular whitish spots and two brownish stripes. The tale is dark-ocher-brownish, with irregular whitish spots and markings. The horizontal yellowish belt-zone of the lower part of the body is distinct. The edge of the V-grove is yellowish-whitish. The sole is uniformly pale. Since all the specimens we found are juveniles, we prefer not to describe a new species. We hope to draw the attention of the scientific community to the area and that future studies will be able to identify and describe this taxon.

### ﻿ Laocaia simovi

Taxon classificationAnimaliaStylommatophoraHelicarionidae

Dedov & Schneppat, 2019

FF913265-5D66-505C-9634-C179552C90E7

Figs 13C, D , 14B1–14 , 3

Laocaia simovi : Dedov and Schneppat 2019, p. 22–29.

#### Material examined.

Vietnam • 1 adult, 1 juvenile; Lào Cai Province, Sa Pa district, Fansipan peak, bamboo forest below the peak; 22.3046°N, 103.7759°E; 3040 m alt.; 24 Oct. 2023; I. Dedov, N. Simov, R. Bekchiev, M. Langurov leg.; IBER-BAS 40619-1.

#### Remarks.

We found color morph of *L. simovi* (Fig. 13D) in 2023 that is more yellowish than the type specimens (Fig. 13C). The 2023 specimens were found very close to the species’ type locality (Fig. 14B). For more details on the species, see Dedov et al. (2019).

### ﻿Key to the genera of slugs and slug-like semi-slugs of the superfamily Helicarionoidea, from North Vietnam

**Table:** 

1	Shell vitrinoid, medium-sized to large (height 5.5–20.0 mm, diameter 11–40 mm), partly covered by mantle lobes	*** Megaustenia ***
–	Shell smaller (width 3.8–6 mm, length 6.3–8.0 mm), greatly reduced to a thin, oval-rhomboid plate, always fully covered by mantle lobes	**2**
2	Epiphallus absent. Bursa copulatrix with well-distinguished spermatheca and pedunculus	*** Laocaia ***
–	Epiphallus present. Bursa copulatrix more or less like a cylindrical tube. Spermatheca and pedunculus not well or weakly defined	**3**
3	Epiphallus without a caecum	*** Muangnua ***
–	Epiphallus with 1–3 caeca	**4**
4	Accessory structures to the genital system missing	*** Myotesta ***
–	Accessory structures (torus-toroid discs or net-like structure) present	*** Ostracolethe ***

## ﻿Discussion

### ﻿Torus structures of *Ostracolethe fruhstorfferi* and metachrosis

The presence of the toroidal structure in *O. fruhstorfferi*, which had first been described by Simroth (1901), indicates that the synonymization of *Ostracolethe* and *Myotesta* by Solem (1966) is unfounded, and that *Ostracolethe* is a valid genus.

Collinge (1902: 14–15), citing Simroth (1901), wrote:

“The most remarkable peculiarity lies in fact that, near point of origin, in adhering muscle, there are a number of plate-shaped discs which can best be likened to buttons, which concave on one side. They are arranged on one base. Each disc has a narrow central, cloven lumen, and arises out of cruciform muscle fibers, of which radiating bundles are enclosed. It is this penis muscle which I wished to bring into connection with attraction and dart-glands of the Vitrinae, which species together with others of Atlantic members of this genus, discharge upon themselves from penis, and must necessarily be more or less permeated by seminal fluid. Although it is quite certain that lumenae of discs must be connected by a canal, I have as yet unfortunately failed to distinguish any such canal in muscular tissue”

We examined the position of torus structures in animals in situ, as well as under magnification, under a binocular microscope in different conditions, unstained and stained and on non-permanent and permanent microscope slides (Fig. 6A–F). We noted that when staining the structure according to Georgiev’s method (1986), acetocarmine was used, which has an acidic reaction, but the discs did not undergo destructive changes, which means that there was no conchiolin or other carbonate elements in them. Judging by the position of the structure in situ in the gastropod body (from one side next to the female reproductive system), we assume that the structure is part of the reproductive system. In Fig. 6C, D, F, we observed the discs producing cells, which move from the central lumen to the surface of the discs where they concentrate and are secreted into the connective tissue cushion and/or the body cavity. We assume that this structure performs a function similar, or is analogous to, the accessory glands in Helicoidea.

From the other side, in situ, tori lie packaged on the esophagus and appear to be connected to the nerve ring (Fig. 5C). This indicates that this structure is under direct (and rapid) control by the nervous system of the animal. In *O. fruhstorfferi* we registered the phenomenon of metachrosis, which is a passive self-defense mechanism for the slug. This phenomenon is known in cephalopod molluscs like octopuses, squids, and cuttlefish, which achieve rapid changes in color using proteins called reflectins (Crookes et al. 2004). These proteins, found in specialized pigment-containing cells called chromatophores, can be rapidly rearranged to alter the way light is reflected, resulting in instantaneous camouflage or display (Crookes et al. 2004; Mathger et al. 2013; Nakajima et al. 2022). In our opinion, most likely the torus structures in *O. fruhstorfferi* enable fast color change and provide protection to the animal. Since there is no definitive evidence for this hypothesis, we plan to continue studying the cellular structure of the tori.

### ﻿Elliptic-net structure of *Ostracolethe* (?) *penevi* Dedov & Vu, sp. nov. and taxonomic position of the species

The elliptical-net structure found in *O. penevi* Dedov & Vu, sp. nov. is the second very unusual one (besides the torus structures of *O. fruhstorfferi*) found among the newly collected specimens, and its function is currently a mystery. It seems to be associated with the female part of the reproductive system, and we assume that it plays a role in reproduction. Since we have a single specimen, which is also the type specimen, we chose not to explore this unusual structure more fully and destroy evidence. The type locality of *O. penevi* Dedov & Vu, sp. nov. is easily accessible, and we plan to search the area again. We hope to collect additional material of this and *O. fruhstorfferi* and to continue to study both unusual structures on a histological and cellular level.

This unusual elliptic-net structure, discovered here for the first time, highly suggests that *O. penevi* belongs to a new genus. However, the lack of sufficient material, the unclear function of the structure, and our reluctance to damage the reproductive system of the type specimen are why we refrain from describing a new genus for now. In the present work, the newly described species is tentatively assigned to the genus *Ostracolethe* due to its plate-like shell, identical structure of the male genitalia (epiphallus with two additional short, uncoiled caeca) (Schileyko 2003), as well as the presence of an unknown structure, potentially belonging to the female part (as in *O. fruhstorfferi*).

## Supplementary Material

XML Treatment for Megaustenia

XML Treatment for Megaustenia cf. messageri

XML Treatment for Ostracolethe

XML Treatment for Ostracolethe fruhstorfferi

XML Treatment for Ostracolethe (?) penevi

XML Treatment for Muangnua

XML Treatment for Muangnua vumanhi

XML Treatment for Laocaia

XML Treatment for Laocaia simovi

## ﻿Additional information

### Use of AI

No use of AI was reported.

### Author ORCIDs

Ivailo Dedov https://orcid.org/0000-0003-4445-359X

Quang Manh Vu https://orcid.org/0000-0002-6561-8392

Tuan Trieu Anh https://orcid.org/0000-0001-5877-2060

### Data availability

All of the data that support the findings of this study are available in the main text.
